# Mutation of the ALS/FTD-associated RNA-binding protein FUS affects axonal development

**DOI:** 10.1523/JNEUROSCI.2148-23.2024

**Published:** 2024-05-01

**Authors:** Francesca W. van Tartwijk, Lucia C.S. Wunderlich, Ioanna Mela, Stanislaw Makarchuk, Maximilian A.H. Jakobs, Seema Qamar, Kristian Franze, Gabriele S. Kaminski Schierle, Peter H. St George-Hyslop, Julie Qiaojin Lin, Christine E. Holt, Clemens F. Kaminski

**Affiliations:** 1 Department of Chemical Engineering and Biotechnology, University of Cambridge, Philippa Fawcett Drive, Cambridge CB3 0AS, UK; 2 UK Dementia Research Institute and Department of Clinical Neurosciences, University of Cambridge, Cambridge Biomedical Campus, Cambridge CB2 OAH, UK; 3 Department of Physiology, Development, and Neuroscience, University of Cambridge, Cambridge CB2 3DY, UK; 4 Cambridge Institute for Medical Research, Department of Clinical Neurosciences, University of Cambridge, Cambridge CB2 0XY, UK; 5 Department of Medicine, University of Toronto and University Health Network and Tanz Centre for Research in Neurodegenerative Diseases University of Toronto, Toronto, ON M5T 0S8, Canada; 6 Taub Institute For Research on Alzheimer’s Disease and the Aging Brain, Department of Neurology, Columbia University Irvine Medical Center, 630 West 168th Street, New York, NY, USA 10032; 7 UK Dementia Research Institute Centre and Institute of Psychiatry, Psychology and Neuroscience, King’s College London, Maurice Wohl Clinical Neuroscience Institute, London, SE5 9NU, UK

## Abstract

Aberrant condensation and localisation of the RNA-binding protein (RBP) fused in sarcoma (FUS) occur in variants of amyotrophic lateral sclerosis (ALS) and frontotemporal dementia (FTD). Changes in RBP function are commonly associated with changes in axonal cytoskeletal organisation and branching in neurodevelopmental disorders. Here, we asked whether branching defects also occur *in vivo* in a model of FUS-associated disease. We use two reported *Xenopus* models of ALS/FTD (of either sex), the ALS-associated mutant FUS(P525L) and a mimic of hypomethylated FUS, FUS(16R). Both mutants strongly reduced axonal complexity *in vivo*. We also observed an axon looping defect for FUS(P525L) in the target area, which presumably arises due to errors in stop cue signalling. To assess whether loss of axon complexity also had a cue-independent component, we assessed axonal cytoskeletal integrity *in vitro*. Using a novel combination of fluorescence and atomic force microscopy, we found that mutant FUS reduced actin density in the growth cone, altering its mechanical properties. Therefore, FUS mutants may induce defects during early axonal development.

## Introduction

The RNA-binding protein (RBP) fused in sarcoma (FUS) forms cytoplasmic aggregates in variants of both amyotrophic lateral sclerosis (ALS) and frontotemporal dementia (FTD) (Kwiatkowski et al., 2009; Neumann et al., 2009; Vance et al., 2009), neurodegenerative diseases that are extremes of one disease spectrum (Ragagnin et al., 2019). In some variants of familial ALS (fALS), FUS’s nuclear localisation sequence (NLS) is mutated, leading to a rise in its cytoplasmic levels followed by aggregation (Vance et al., 2013). In classical FTD, unmutated FUS becomes cytoplasmic upon arginine residue hypomethylation (Dormann et al., 2012). However, the pathological importance of cytoplasmic mislocalisation versus subsequent aggregation remains incompletely understood.

As mutated FUS is expressed during development in fALS patients, changes in axonal biology could occur prior to degeneration onset. FUS is known to be essential for development from mouse studies: its knock-out results in perinatal lethality (Hicks et al., 2000) and splicing changes in the central nervous system (Humphrey et al., 2020), but post-natal knockout does not cause motor neuron death (Sharma et al., 2016). Notably, homozygous knock-in of FUS(ΔNLS) also results in loss-of-function splicing defects and perinatal lethality (Humphrey et al., 2020), indicating FUS’s nuclear roles are important. However, homozygous FUS(ΔNLS) mice show pre-birth motor neuron apoptosis, unlike knock-out mice (Scekic-Zahirovic et al., 2016), indicating cytoplasmic mislocalisation also causes toxic gain of function (Scekic-Zahirovic et al., 2016). As FUS mutants are not perinatally lethal when heterozygous, it is less clear whether wild-type FUS expression can mitigate this developmental toxicity. However, there are indications that neurodevelopment remains affected: paediatric ALS patients carrying *fus* NLS mutations may present initially with learning disabilities, tremor, and mild motor developmental delay (Picher-Martel et al., 2020), and heterozygous FUS(ΔNLS) mice likely display developmental dendritic excitability changes (Sahadevan et al., 2021).

Potential effects of mutant FUS on axonal morphogenesis are of particular interest in understanding this developmental toxicity. Axon branching is critical for establishment of neuronal connectivity and is compromised in a range of neurodevelopmental disorders, including due to changes in axonal protein synthesis (Lin et al., 2021), which FUS is known to affect (Murakami et al., 2015; López-Erauskin et al., 2018; Qamar et al., 2018). There are indications in the literature supporting the hypothesis that mutant FUS may compromise developmental axonal branching. The effects of NLS FUS mutants on branching have been studied on unguided neurites *in vitro*, with different results: branching was decreased by FUS(R521C) in primary cortical axons and dendrites and in motor neuron dendrites (Groen et al., 2013; Qiu et al., 2014), but increased by FUS(H517D) or FUS(P525L) in human iPSC-derived motor neuron axons (Akiyama et al., 2019; Garone et al., 2021). However, axon morphogenesis is heavily regulated by local guidance and trophic cues *in vivo* (Cioni et al., 2018). Changes in these cues occur in (pre)symptomatic ALS mouse models and in sporadic ALS patients (Dupuis et al., 2002; Jiang et al., 2005; De Winter et al., 2006; Schmidt et al., 2009; Krakora et al., 2012; Moloney et al., 2014). Therefore, the effects of mutant FUS on branching should be studied further in developing axons *in vivo*. There is also evidence that *fus* mutation affects the axonal cytoskeleton, the remodelling of which is critical for branching (Nanda et al., 2020; Bodakuntla et al., 2021): mutant FUS(P525L) may downregulate translation of some cytoskeletal proteins (Garone et al., 2020), FUS(R495X) aggregation reduces the number of detyrosinated microtubules (Yasuda et al., 2017), and FUS(R521C) aggregates sequester the actin-associated *nd1-L* mRNA (Jun et al., 2017).

Here, we therefore sought to study FUS-associated cytoskeletal and branching changes in a developmental axonal model system. We used two previously established ALS/FTD *Xenopus laevis* models (Murakami et al., 2015; Qamar et al., 2018): the ALS-associated NLS mutant FUS(P525L), a common cause of juvenile ALS (Picher-Martel et al., 2020), and an artificial mimic of hypomethylated FUS, FUS(16R), which contains sixteen strategically inserted arginine residues to increase the protein’s overall hypomethylation state (Qamar et al., 2018).

## Materials and methods

### Software availability

All data reported in this paper and custom-written MATLAB code for quantitative fluorescence microscopy will be shared by the lead contact (CFK) upon request. Any additional information required to reanalyse the data reported in this paper is available from the lead contact upon request.

### Experimental model and subject details

#### *Xenopus laevis* embryos

*X. laevis* eggs were fertilised *in vitro*. Embryos were raised in 0.1x modified Barth's saline (MBS) (8.8 mM NaCl, 0.1 mM KCl, 0.24 mM NaHCO_3_, 0.1 mM HEPES, 82 μM MgSO_4_, 33 μM Ca(NO_3_)_2_, 41 μM CaCl_2_) at 14-18°C and staged according to the tables of Nieuwkoop and Faber (1994). This research has been regulated under the Animals (Scientific Procedures) Act 1986 Amendment Regulations 2012 following ethical review by the University of Cambridge Animal Welfare and Ethical Review Body (AWERB).

#### Primary *Xenopus* retinal cultures

RGC culture was performed as described by Leung and Holt (2008). In brief, once embryos reached stage 33-34, eye primordia were dissected from MS222 (Merck)-anaesthetised embryos of either sex and placed in No. 1.5 glass-bottom dishes (MatTek) that had been pre-coated overnight with poly-L-lysine (10 μg/mL, Merck) and subsequently for 1 h with laminin (10 μg/mL in L15 medium, Merck). RGC cultures were kept in 60% L15 medium with 1X Antibiotic-Antimycotic (Gibco) at 20°C. Imaging was performed after overnight axon outgrowth. 3-4 eye primordia were cultured per condition per dish and, typically, 2-3 dishes were used per experimental condition for each biological replicate. Replicates in each experiment using *X. laevis* in this study were obtained from different batches of embryos.

## Method details

### Construct expression

To generate RGCs expressing different FUS variants of interest, fused to GFP, *X. laevis* embryos were injected with *in vitro* synthesised mRNA. At the four-cell stage, blastomeres that will form the dorsal and ventral halves of the embryo are distinguishable, and injection of both dorsal blastomeres results in mRNA translation in both brain and eye tissue. Injected mRNAs encoded GFP fused to full-length wild-type (WT) FUS, FUS(P525L), or FUS(16R). A GFP-only control was also included. Injection was performed as described by Leung and Holt (2008). In brief, capped and polyadenylated mRNA was synthesised *in vitro* from plasmid stocks (Das et al., 2003; Qamar et al., 2018) using the mMESSAGE mMACHINE™ SP6 transcription and poly(A) tailing kits (Invitrogen). This was subsequently diluted to a standard concentration of 200 ng/μL (100 ng/μL for GFP mRNA). Both dorsal cells of embryos at the four-cell stage were injected with 5 nL of mRNA solution.

Prior to every experiment, embryos were screened for fluorescence at stage 28 (for *in vivo* experiments involving electroporation) or stage 33/34 (for *in vitro* experiments involving RGC culture) using a home-built microscope featuring an IX81 frame (Olympus), an LED light source (Thorlabs), an MDF-WGFP filter set (Thorlabs; excitation 445 ± 22.5 nm and emission 510 ± 21 nm), and a 10X/0.25 NA air objective; only embryos that were GFP-positive throughout the head and in the spine were included in experiments.

### Western blotting of *X. laevis* head lysates

Heads from stage 39/40 embryos were collected (15 per condition) and homogenised by pipetting in Pierce RIPA buffer (Thermo Fisher Scientific) supplemented with Pierce protease inhibitor (Thermo Fisher Scientific). Homogenised samples were further lysed for thirty minutes on ice, then frozen overnight at 80°C. After thawing on ice, samples were spun at 21,100 g for 15 minutes at 4°C, and the supernatant was collected. Protein concentration was determined using a Pierce BCA protein assay kit (Thermo Fisher Scientific). For gel electrophoresis, a lysate volume corresponding to 20 μg of total protein was used for each condition, and combined with NuPAGE LDS sample buffer (Invitrogen) supplemented with β-mercaptoethanol. The lysate-dye mixture was boiled at 95°C for 5 min and then loaded onto a 4-12% NuPAGE gel, which was run at 150 V for 90 minutes using an Invitrogen system. A Spectra™ multicolor broad range protein ladder (Thermo Fisher Scientific) was also included. Proteins were then transferred onto a PVDF transfer membrane (Thermo Fisher Scientific) at 30 V for 90 min. The membrane was subsequently blocked for 1 h at room temperature in 5% skim milk powder (Merck) in PBS. The membrane was then cut at the 50 kDa ladder marker to allow separate staining with a rabbit anti-FUS antibody (AV40278, Merck; 1:1000 diluted) and a mouse anti-GAPDH antibody (G8795, Merck; 1:1000 diluted). Membrane halves were incubated with primary antibody overnight at 4°C, and subsequently washed three times for 5 min with 0.1% Tween20 in PBS. Membrane halves were then incubated for 1h at room temperature with ECL secondary antibody (anti-mouse NA934V and anti-rabbit NA931VS, Merck; both 1:2500 diluted). After three washes with 0.1% Tween20 (Merck) in PBS, membranes were incubated for 1 min with SuperSignal West Pico PLUS chemiluminescent substrate (Thermo Fisher Scientific) and then imaged using a Syngene G:box.

### Quantitative fluorescence microscopy

RGC cultures were fixed with 2% formaldehyde and 7.5% sucrose in PBS for 20 min at room temperature. The samples were washed 5 times with PBS and permeabilised with 0.1% Triton-X-100 in PBS for 5 min followed by 3 more washing steps with PBS. After blocking of samples with 5% donkey serum in PBS for 1 h, mouse anti-β-tubulin antibodies (ab131205, Abcam) diluted 1:300 in blocking solution were applied and samples incubated overnight at 4°C. Dishes were washed with PBS 5 times for 3 min each before applying donkey anti-mouse Alexa Fluor 568 secondary antibodies (A10037, Invitrogen; 1:2000 diluted) and Alexa Fluor 647 phalloidin (A22287, Invitrogen; 5 u/mL) in blocking solution for 1 h. Samples were washed again with PBS 5 times for 3 min each before imaging on a home-built widefield microscope. The microscope frame (IX83, Olympus) is equipped with an LED light source (DC4100, Thorlabs), a sCMOS camera (Zyla 4.2, Andor) and is controlled with the software Micro-Manager (Open Imaging). All images were acquired with a 60×/1.42 oil objective lens (PlanApoU, Olympus).

### RNA granule tracking

To visualise RNA granule dynamics, cultured RGC axons were imaged under a Perkin Elmer Spinning Disk UltraVIEW ERS, Olympus IX81 inverted microscope with a 60x 1.4NA silicone oil objective. One-minute movies of axons were acquired (two frames per second, at constant exposure time and laser intensity).

### Atomic force microscopy

AFM measurements were performed using a commercial Bioscope Resolve atomic force microscope (Bruker, Santa Barbara). For the mechanical measurements, pre-calibrated Live Cell s (PFQNM-LC, Bruker AFM probes) cantilevers were used, with a nominal spring constant of 0.07 N/m s and the deflection sensitivity was measured at the start of each experiment using no touch calibration. The nominal radius of the probe is 70 nm. The microscope was operated in PeakForce QNM mode, data sampling rate was 0.25 kHz in most experiments and the force setpoint was selected so that the maximal indentation depth was around 200 nm. For treatment with Cytochalasin-D, 10 μg/mL of Cytochalasin-D was applied for 30 minutes prior to measurement.

### Branching experiments

For branching experiments, injected embryos were electroporated at stage 28 (Wong and Holt, 2018). Electroporation was performed with a plasmid encoding mCherry (pCS2+ backbone). Two drops of solution (1 μg/μL) were delivered to the right eye primordium, quickly (<3s) followed by two pulses of 18 V (pulse width 50 ms, interval 1000 ms). This sparsely introduces the plasmid into RGCs, resulting in a small number of labelled axons. Branching axons in the optic tectum were imaged using the 60X oil objective on the same custom-built widefield microscope used for quantitative fluorescence microscopy experiments, as z-stacks with a spacing of 0.5 μm. To this purpose, the left (unelectroporated) eye and skin on the brain were removed, and embryos were mounted on permanox slides (Thermo Fisher Scientific) in frame-seal incubation chambers (Bio-Rad).

### Experimental design and statistical analyses

#### Statistics

For all experiments, the *N*-number, number of biological replicates, and the statistical tests applied are described in the figure legends. Statistical significance is defined as: n.s. for not significant, * for p < 0.05, ** for p < 0.01, *** for p < 0.001, and **** for p < 0.0001. Statistical analysis was performed using Python 3.8.1 in Visual Studio Code.

For statistical analyses for each experiment, data from different replicates were pooled, with each observation from each replicate being used as a datapoint. (Experiments here refers to branching, quantitative fluorescence microscopy, AFM, and RNA granule tracking experiments; replicates here refers to experiments performed on different days using material from different batches of embryos.) For each experiment, variance within replicates was large for each condition. This precluded the use of analyses using (weighted) averages of replicates as datapoints. As results from different replicates were comparable, datapoints from all replicates were subsequently pooled for analyses. Therefore, the total number of observations per condition was used as the *N*-number.

#### Quantitative fluorescence microscopy

For quantitative fluorescence analysis, a custom-written MATLAB code was used to select individual growth cones. Only morphologically normal (non-collapsed) single growth cones were included in this analysis, to avoid selection bias. After background subtraction, total intensity values were normalised to the signal area (i.e., the whole growth cone for actin staining, and the central region only for tubulin staining). Masks defining the area of interest were generated computationally following thresholding for actin, and by manual outlining for tubulin using Fiji (Schindelin et al., 2012).

#### RNA granule tracking

Kymographs were generated in Fiji (Schindelin et al., 2012), with a constant linewidth of 20 pixels. Granule paths were automatically detected and analysed using the KymoButler software (Jakobs et al., 2019). Distal ends of axons were imaged to enable imaging of unbundled and non-crossing axons, as well as unambiguous identification of anterograde and retrograde direction of motion in subsequent analysis (by presence of the growth cone).

#### Atomic force microscopy

At least five cells per condition were measured and at least 10 force curves per cell per condition were analysed. Force curves were analysed only for areas of a height of >150 nm. The extension part of the force curves (5 to 20% of the indentation) was fitted to a linearised Hertz model using Nanoscope 9.1 (Bruker), from which the Young’s modulus corresponding to each force curve was calculated.

Force curve fitting using a linearised Hertz model requires two assumptions to be met in order for the results to solely represent the Young’s modulus. This model assumes (1) that the sample’s thickness is infinite and (2) that the sample’s response to deformation is in the linear elastic regime (Dimitriadis et al., 2002). If indentation is more than ten percent of sample height, the first assumption does not hold, and substrate effects contribute to the calculated apparent stiffness (Domke and Radmacher, 1998). This is the case for these measurements, but as height was comparable for growth cones expressing different FUS mutants, it does not prevent a comparative rather than absolute study of the effects of FUS on cellular mechanical properties. If narrow probes are used to apply force, deformation is not in the low-strain regime, and the second assumption does not hold (Dimitriadis et al., 2002). Here, conical probes with spherical tips of radius 70 nm were used. Therefore, the observed response does not solely reflect the Young’s modulus of sample and substrate, but also non-linear sample responses to high local strain. Therefore, these AFM measurements represent changes in cellular mechanical properties, rather than direct measurements of cell stiffness (Young’s modulus), and are reported as the apparent Young’s modulus.

#### Branching analysis

All axons that were wholly and unambiguously traceable were included in the analysis (where up to three were labelled, or one or two axons were much brighter than other axons). As plasmid is occasionally electroporated into brain or spine cells, only axons within the correct orientation in the optic tectum were included in analyses. Analysis was performed using the SNT Neuroanatomy plugin for Fiji (Schindelin et al., 2012; Arshadi et al., 2021). Branch order was defined so that the number of higher-order branches was minimised (e.g., for a primary branch containing a branch point, splitting into branches A and B: if A had another branch point, but B did not, A would be considered the continuation of the primary branch, rather than B, which would be a secondary branch). Where two daughter branches had an equal number of branch points, branch order was defined so that higher-order branch length was minimised (e.g., for a primary branch containing a branch point, splitting into terminal branches A and B: if A were longer than B, A would A would be considered the continuation of the primary branch, rather than B, which would be a secondary branch). The ACI was calculated for each axon as the weighted fraction of higher-order branches: *ACI* = ∑_*i*_ *iN*_*i*_/∑_*i*_ *N*_*i*_ (where *i* is branch order and *N* branch number) (Marshak et al., 2007).

## Results

**Figure 1:**
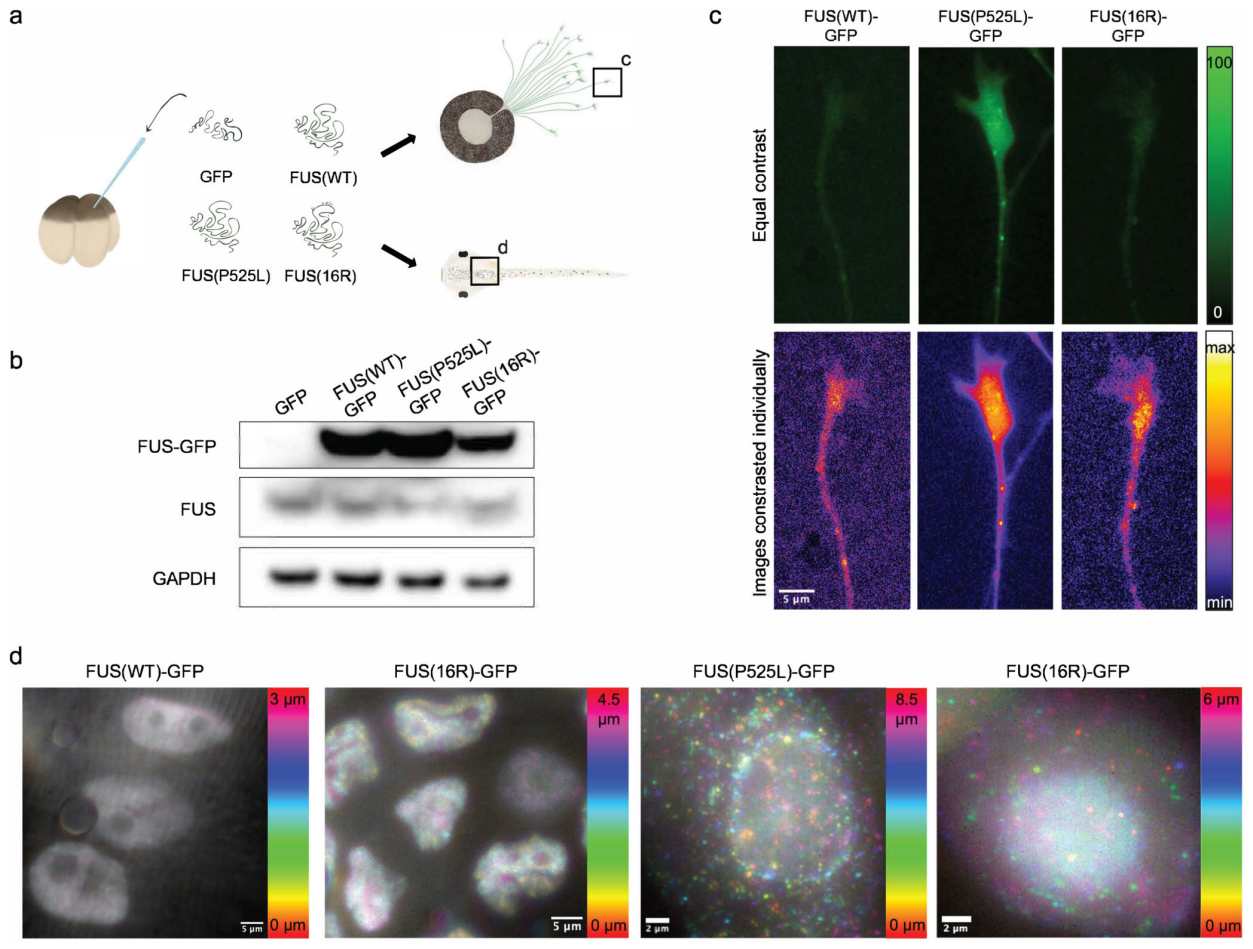
The expression and localisation of GFP-tagged FUS constructs is as expected. a) Embryos were injected with (FUS-)GFP-encoding mRNA at the four-cell stage. Construct localisation was imaged in cultured RGCs and whole embryos. b) Exogenous FUS expression does not result in loss of endogenous FUS. Western blotting was performed on whole-head lysates from stage 39/40 embryos. c) FUS-GFP constructs localise correctly in cultured RGC axons. Signal intensity is higher for FUS(P525L)-GFP. Occasional granules are apparent for all FUS constructs. d) FUS-GFP constructs localise correctly *in vivo*. FUS(WT) is nuclear and excluded from nucleoli. FUS(16R)-GFP is largely nuclear, but cytoplasmic granules can be observed. FUS(P525L)-GFP has a large cytoplasmic fraction, and granules can be observed.

To generate *Xenopus* embryos expressing different FUS constructs, embryos at the four-cell stage were injected with mRNA encoding C-terminally GFP-tagged wild-type (WT) FUS, FUS(P525L), or FUS(16R) (Figure 1a). Expression of these constructs did not cause developmental delays or morphological defects. A GFP-encoding mRNA condition was also included as a control for any effects of exogenous FUS expression. As an additional control, we tested the effects of the different human FUS variants on endogenous FUS expression. As determined by Western blotting, mRNA injection did not cause loss of endogenous FUS expression (Figure 1b). While limited cross-reactivity of anti-human FUS antibodies prevented direct comparison of human and endogenous FUS expression levels, this experiment also demonstrated that FUS(WT)-GFP and FUS(P525L)-GFP are expressed at comparable levels, while FUS(16R)-GFP appears to be degraded at a slightly higher rate (Figure 1b). Therefore, any phenotypic effects of mutant FUS are not due to higher total FUS levels relative to the FUS(WT)-GFP condition, or due to changes in endogenous FUS levels, but are specific to the expression of mutant variants. We also confirmed our protein constructs persisted throughout the developmental period studied: fluorescent protein expression was readily detectable up to stage 45 throughout the head and spine (ventral) side of the embryo. To further confirm GFP tagging did not affect FUS function, we validated the correct localisation of the FUS-GFP constructs in cultured RGC axons at stage 35/36 (Figure 1c) and *in vivo* at stage 45 (Figure 1d): FUS(WT)-GFP and FUS(16R) were largely nuclear, with some granules being present in axons, to a greater extent for FUS(16R) (Qamar et al., 2018); in contrast, FUS(P525L)-GFP mislocalised to the cytoplasm to a significant extent.

### Mutant FUS compromises axonal branching

**Figure 2:**
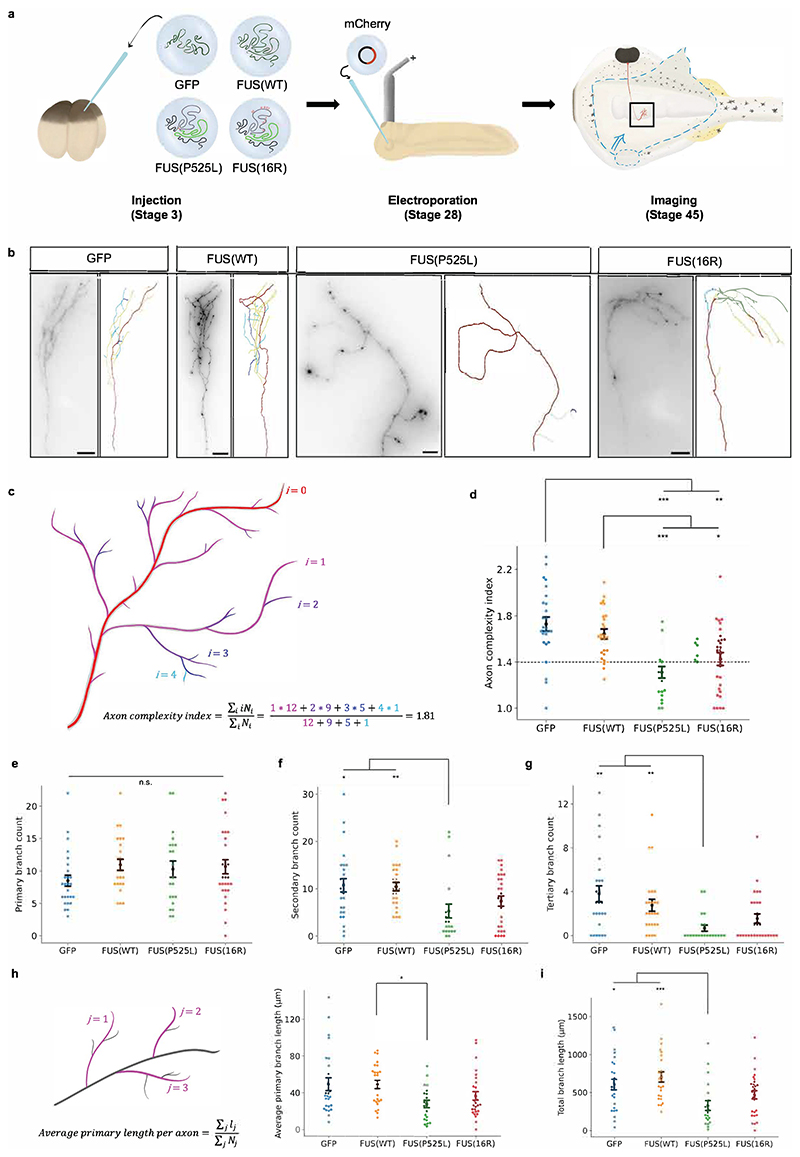
Mutant FUS compromises axonal branching. a) Schematic of experimental procedure. mRNA encoding only GFP (green) or FUS (grey) fused to GFP is injected at the four-cell stage. At stage 28, axons are sparsely labelled by electroporation, which are then imaged at stage 45. b) Sample images of mCherry-labelled axons expressing different GFP or FUS-GFP constructs. c) Schematic of calculation of axon complexity index (ACI). d) Mutant FUS reduces the axon complexity index. Dashed line indicates value below which axons are considered ‘simple’. e-g) FUS(P52L) reduces the number of higher-order branches. Plots show number of primary, secondary, and tertiary branches per axon respectively. h) FUS(P525L) may reduce average primary branch length. i) FUS(P525L) reduces total branch length. d-i): Number of axons analysed: n_GFP_=26; n_FUS(WT)_=25; n_FUS(P525L)_=21; n_FUS(16R)_=28. N≥6 replicates for each condition. All conditions were compared pairwise, those that are significantly different are indicated. Kruskal-Wallis tests with Bonferroni correction, error bars indicate standard errors in means.

To investigate the effects of mutant FUS on axon organisation in a physiological context, we visualised RGC axon branching *in vivo* (Figure 2a). At stage 28, when the eye primordium is identifiable, this tissue was electroporated with an mCherry-encoding plasmid, selectively labelling RGC axons but not brain tissue (Wong and Holt, 2018). At stage 45, when the axonal arbour has stabilised, embryo brains were exposed to image RGC axons within the optic tectum (their target area).

Expression of both FUS(P525L)-GFP and FUS(16R)-GFP reduced axonal complexity compared with GFP-only and FUS(WT)-GFP controls (Figure 2b). Morphological complexity was quantified using the axon complexity index (ACI), a measure of the fraction of higher-order axonal branches (Marshak et al., 2007) (Figure 2c). The average ACI of stage 45 *X. laevis* RGCs is in the 1.8 to 2.0 range, with an axon with ACI<1.4 being designated simple (Wong et al., 2017; Cagnetta et al., 2019; Shigeoka et al., 2019). The ACI was significantly lower for axons expressing FUS(P525L) and FUS(16R) compared to GFP or FUS(WT) (Figure 2d). The decrease in ACI associated with FUS(P525L)-expression was due to a decrease in the number of secondary and tertiary branches, as there was no reduction in primary branch numbers (Figure 2e-g). However, the average length of primary axon branches appeared to be reduced for FUS(P525L) (Figure 2h), indicating that defects occur already at the primary branching stage. The overall branch length was also reduced for FUS(P525L)-expressing axons compared with both GFP and FUS(WT)-GFP controls (Figure 2i).

### FUS(P525L) causes developmental transition defects

**Figure 3:**
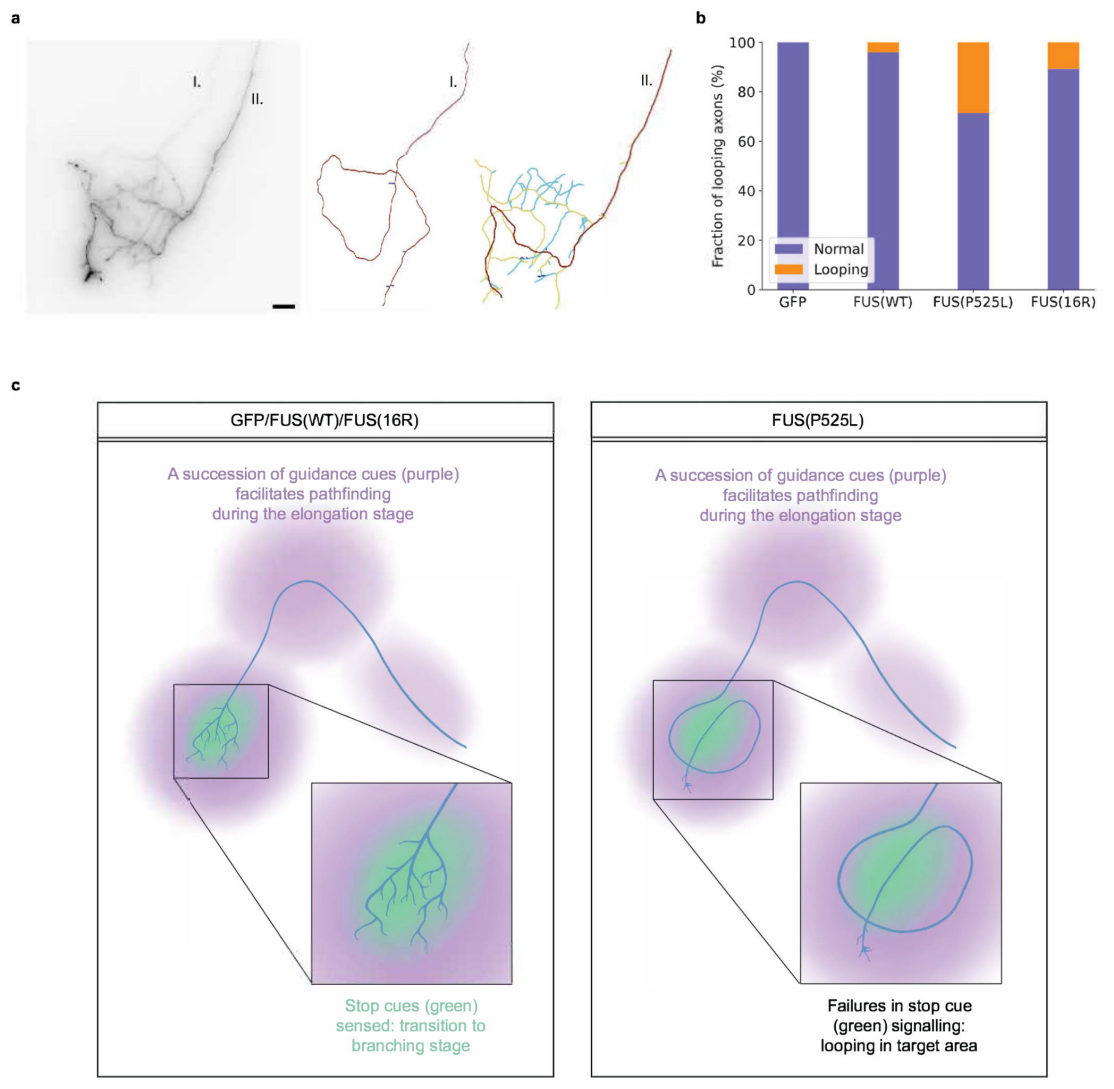
FUS(P525L) causes axon ‘looping’. a) Image of looping axon and normal axon within the brain of a FUS(P525L)-expressing embryo (scale bar: 20 μm). b) Looping is more common in FUS(P525L)-expressing axons. (Fisher’s exact test of FUS(P525L) vs GFP and FUS(WT): p=0.005 and p=0.03 respectively. Number of axons analysed: n_GFP_=26; n_FUS(WT)_=25; n_FUS(P525L)_=21; n_FUS(16R)_=28.) c) Looping can be explained as a developmental transition defect.

Notably, a subset of FUS(P525L)-expressing axons displayed an aberrant ‘looping’ phenotype (Figure 3a-b). While these axons reached the optic tectum without apparent errors in navigation to this target area, the main axon shaft executed an almost complete turn or loop within the tectum. We defined axons as ‘looping’ if they displayed apparent turning behaviour and were simple (ACI<1.4). Looping was rarely seen for axons expressing GFP, FUS(WT), or FUS(16R) (Fisher’s exact test of FUS(P525L) vs GFP or FUS(WT): *p*=0.005 and *p*=0.03 respectively).

This looping phenotype represents a failure of the axon to exit the elongation stage, which is a defect in “stop cue”-dependent axon remodelling (Figure 3c). In *X. laevis* RGCs, the growth cone halts advancement when it reaches the optic tectum, and does not overshoot its target area (Harris et al., 1987). Branching then occurs through the formation of filopodia at or near the base of the growth cone, notably also in axons that have been recently severed, and therefore independently of material from the soma (Harris et al., 1987). A defect in this pausing process would result in looping: the axon is able to sense navigational guidance cues, and so is confined to the tectum upon arrival, but is unable to exit the advancement phase, resulting in looping growth within the defined volume of the tectum. Since such axons do not properly enter the branching stage, they display a striking reduction in branch numbers compared with non-looping axons and remain ‘simple’. The halt to extension of axons that reach their target area is thought to be mediated by specific stop cues (Cornel and Holt, 1992), and so signalling through these may be specifically altered (as opposed to signalling via guidance cues generally). Therefore, these data together suggest that while both FUS(16R) and FUS(P525L) affect the developing axon, FUS(P525L) may perturb specific signalling processes that guide axonal arborisation, namely the response to stop cues (or their availability).

### Mutant FUS consistently compromises the growth cone but not the axonal cytoskeleton

We next tested whether mutant FUS also causes cue-independent defects in the basal ability of axons to support the localised polymerisation of cytoskeletal filaments, as this is at the basis of axonal branching. In particular, given that we observed no axon looping for FUS(16R), we sought to determine whether there was a more prominent cue-independent defect in this mutant. The formation of branches along the axon shaft has been shown to occur in the same three general steps as axon outgrowth (protrusion, engorgement, consolidation) (Dent and Gertler, 2003), which are mediated through the dynamic migration and cytoskeletal reorganisation of growth cones (Omotade et al., 2017). Consequently, studying the growth cone’s cytoskeleton can provide a level of information on the axon’s ability to form branches. Therefore, we studied RGC growth cone behaviour *in vitro*, in the absence of cues.

**Figure 4:**
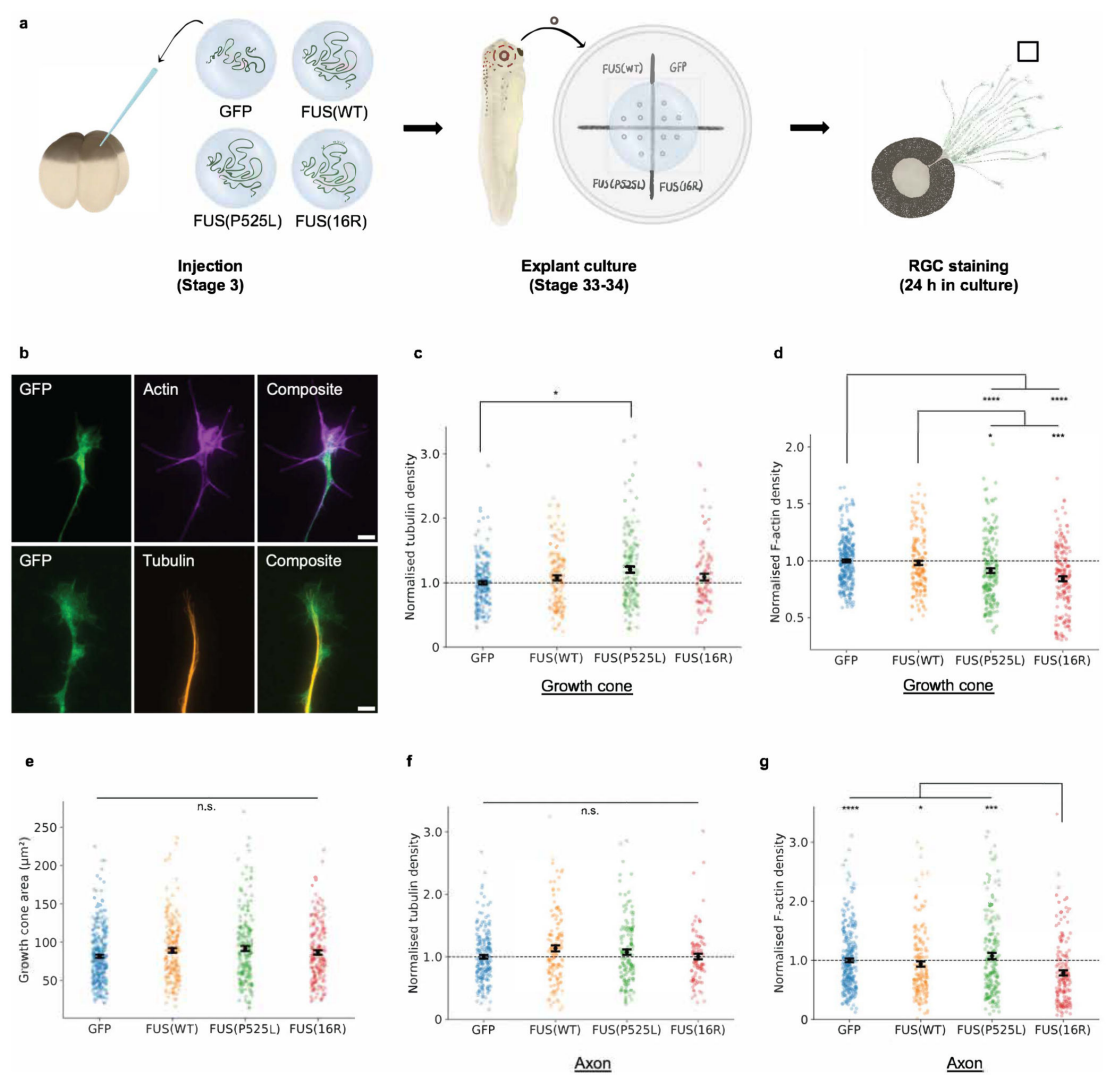
Mutant FUS expression affects growth cone cytoskeletal filament density. a) Schematic of experimental procedure. Embryos are injected as previously. Eye primordia are cultured at stage 33-34, and RGCs are fixed and stained after overnight outgrowth. Fluorescent signal is then quantified. b) Sample images of growth cones. For quantitative imaging, growth cones are labelled with phalloidin (staining F-actin) or an anti-β-tubulin antibody (staining tubulin) (scale bar: 5 μm). c) FUS(P525L) may increase growth cone normalised microtubule density. (Number of growth cones analysed: n_GFP_=204; n_FUS(WT)_=139; n_FUS(P525L)_=139; n_FUS(16R)_=96.) d) Mutant FUS reduces normalised F-actin intensity in growth cones. (Number of growth cones analysed: n_GFP_=309; n_FUS(WT)_=182; n_FUS(P525L)_=197; n_FUS(16R)_=182). e) Growth cone area is not affected by expression of mutant FUS. (Number of growth cones analysed as in Figure 4d). f) Normalised axon shaft tubulin density is not affected by mutant FUS. (Number of axons analysed: n_GFP_=201; n_FUS(WT)_=137; n_FUS(P525L)_=143; n_FUS(16R)_=99.) g) Normalised axon shaft F-actin density is affected by FUS(16R) but not FUS(P525L). (Number of axons analysed: n_GFP_=285; n_FUS(WT)_=171; n_FUS(P525L)_=188; n_FUS(16R)_=170.) (c-g): N≥3 replicates for each condition. All conditions were compared pairwise, those that are significantly different are indicated. Kruskal-Wallis tests with Bonferroni correction, error bars indicate standard errors in means.

As a first test of baseline cytoskeletal organisation, we quantified the density of cytoskeletal filaments in the growth cone under non-stimulated conditions. We immunostained filamentous actin (F-actin) with phalloidin and microtubules with anti-β-tubulin in cultured RGCs and quantified filament densities from fluorescence microscopy images (Figure 4a). As for *in vivo* experiments, analyses were performed on RGCs expressing GFP, FUS(WT)-GFP, FUS(P525L)-GFP, or FUS(16R)-GFP following mRNA injection. As expected, microtubules were largely confined to the central region of the neuronal growth cones, whilst F-actin was dense in the peripheral region (Omotade et al., 2017) (Figure 4b).

**Figure 5:**
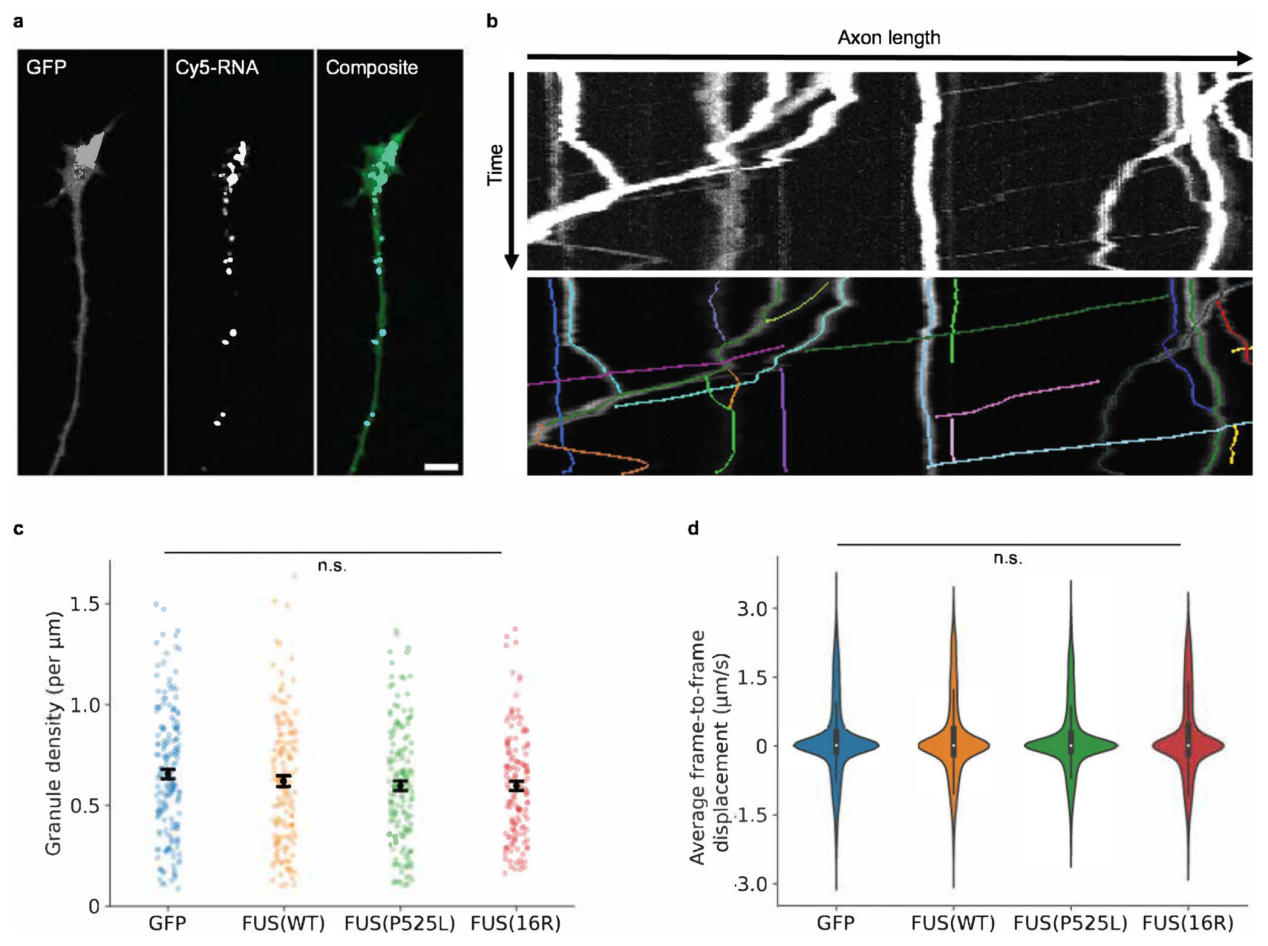
FUS expression does not alter axonal RNA transport dynamics. a) Sample preparation procedure. mRNA is co-injected with Cy5-UTP, which becomes incorporated into all embryonically synthesised RNA. RGCs are cultured as previously. Cy5-labelled RNA is visible as granules in the axon and growth cone (Piper et al., 2015; Wong et al., 2017). b) Sample image of RNA granules in GFP-expressing RGC axon (scale bar: 5 μm). c) Sample kymograph and track detection by KymoButler (Jakobs et al., 2019). d) Average granule density per unit length for individual axons is not altered by mutant FUS expression. (Total number of axons analysed: n_GFP_ = 180; n_FUS(WT)_ = 138; n_FUS(P525L)_ = 155; n_FUS(16R)_ = 136.) e) Average frame-to-frame displacement for individual granules in 60 s is not altered by mutant FUS expression.(Total number of tracks identified: n_GFP_ = 8587; n_FUS(WT)_ = 6710; n_FUS(P525L)_ = 7609; n_FUS(16R)_ = 6202) d-e) N=3 replicates for each condition. Kruskal-Wallis tests with Bonferroni correction, error bars indicate standard errors in means.

Using this method, we observed differences in cytoskeletal density in the growth cone for both mutants. Growth cone tubulin density was increased by FUS(P525L) relative to GFP, but not by FUS(16R) (Figure 4c). In contrast, both FUS mutants caused changes in RGC actin density. Growth cone F-actin density was significantly reduced upon expression of FUS(P525L) and FUS(16R) compared with FUS(WT) and GFP-only controls (Figure 4d). This reduction was similar for both mutants. As growth cone area was unaltered (Figure 4e), this density change represents a decrease in total growth cone F-actin rather than a morphological change. Therefore, FUS(P525L) and FUS(16R) both affect the growth cone’s cytoskeleton, particularly the actin cytoskeleton. The axon shaft was not similarly affected by both mutants, with no changes in F-actin or tubulin density being observable for FUS (P525L), and a reduction in F-actin density only for FUS(16R) (Figure 4f-g). Consistently, axonal RNA transport (which is microtubule-dependent (Lin et al., 2021)) was unaffected by mutant FUS (Figure 5).

### Cytoskeletal defects in mutant FUS-expressing growth cones alter mechanical properties

**Figure 6:**
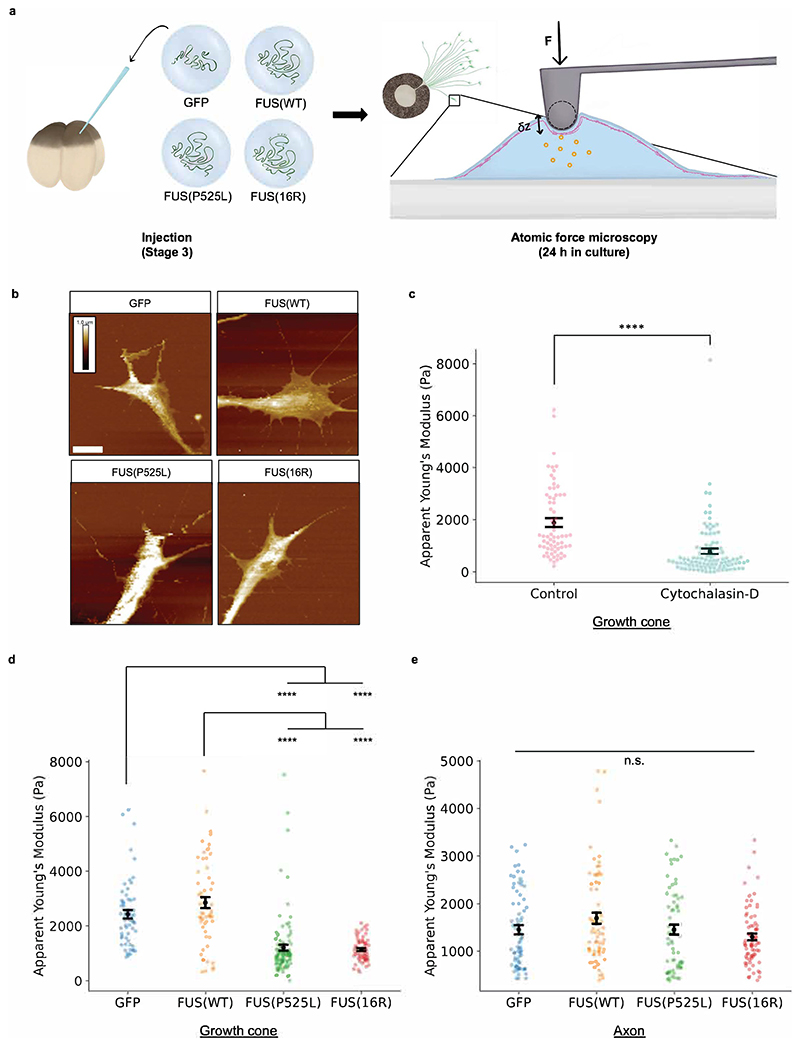
Mutant FUS compromises growth cone mechanoproperties. a) Schematic of experimental procedure. Embryos are injected and RGCs are cultured as previously (Figure 4a). After outgrowth, force-displacement curves are measured for different parts of individual growth cones b) Sample height maps of growth cones. Scale is the same for all images and shown for GFP-only condition: height is colour-coded, white scale bar (bottom left) is 5 μm. c) Cytochalasin-D treatment lowers the apparent Young’s modulus of growth cones. (Number of force curves analysed: n_Control_=66; n_Cytochalasin-D_=100.) d) The apparent Young’s modulus of growth cones is reduced by mutant FUS expression. (Number of force curves analysed: n_GFP_=59 from 5 cells; n_FUS(WT)_=61 from 5 cells; n_FUS(P525L)_=104 from 9 cells; n_FUS(16R)_=64 from 5 cells.) e) The apparent Young’s modulus of axon shafts is not affected by mutant FUS expression. (Number of force curves analysed: n_GFP_=71 from 5 cells; n_FUS(WT)_=64 from 5 cells; n_FUS(P525L)_=69 from 5 cells; n_FUS(16R)_=75 from 5 cells.) c-e) N≥3 replicates for each condition. All conditions were compared pairwise, those that are significantly different are indicated. Kruskal-Wallis tests with Bonferroni correction, error bars indicate standard errors in means.

Our fluorescence data indicate that mutant FUS reduces F-actin density in the growth cone, which we sought to confirm with a more sensitive method. This F-actin change would strongly affect the growth cone’s Young’s modulus: it is known that the dense actin network of the growth cone contributes considerably to the local stiffness (Xiong et al., 2009), in a manner than depends non-linearly on its density as well as other features like its degree of crosslinking (Gardel et al., 2004; Pegoraro et al., 2017; Chen et al., 2020; Wang et al., 2020), while the stiffness of axon shafts is dominated by their microtubules (Ouyang et al., 2013). Therefore, we next developed an approach to measure the mechanical properties of growth cones and axon shafts, using atomic force microscopy (AFM) (Figure 6a). As growth cones are very thin and soft samples, we restricted our measurements to the thicker areas to minimise substrate effects, of which heights were similar between conditions Figure 6b). To validate this approach could detect changes in mechanical properties upon changes in actin organisation, cultures were subjected to brief treatment with cytochalasin D. This drug inhibits F-actin polymerisation and therefore disrupts the F-actin network (Cooper, 1987; Piper et al., 2015). This resulted in the expected decrease in apparent Young’s modulus (Figure 6c).

Using this methodology, we established that both FUS mutants affected the mechanoproperties of growth cones, but not those of axons (Figure 6d-e). This reduction in growth cone but not axon apparent Young’s modulus is consistent with our fluorescence data, demonstrating that changes in growth cone actin occur without changes in axon shaft microtubules. Data distributions were similar for different growth cones and axons with the more sensitive AFM technique, indicating the majority of or all axons expressing mutant FUS are affected. This change in the axon’s ability to support dynamic actin polymerisation is consistent with a loss of ability to support branch initiation, and indicates there is an axon-autonomous component to the observed branching phenotype.

## Discussion

In both neurodevelopmental and neurodegenerative disorders, dysfunction of synapses is central to pathology (Lin et al., 2021), but this has been considered to occur at different life stages. In this categorisation, neurodevelopmental disorders result from failure in the *establishment* of synaptic connectivity (Bagni and Zukin, 2019), while *loss* of normally established synapses occurs in patients with neurodegenerative disorders, correlating with cognitive decline (Selkoe, 2002; Mallucci, 2009; Milnerwood and Raymond, 2010; Serrano-Pozo et al., 2011). However, recent insights reveal that certain proteins associated with neurodegeneration also play roles in neurodevelopment and synaptogenesis (Schor and Bianchi, 2021). This leads to the hypothesis that synaptic function may be compromised early on, or even during development, in neurodegenerative disorders (Taoufik et al., 2018). This would be particularly relevant in the case of familial variants of disease, including of ALS.

The mutant FUS-induced changes in axonal arborisation we observe have implications for a range of critical axonal functions, including synaptogenesis. Axon branching and synaptogenesis are linked developmental processes: synaptogenesis both defines branch initiation sites and stabilises branches (Meyer and Smith, 2006; Ruthazer et al., 2006). This is functionally important: increased branch formation increases axonal area, fostering high local densities of synapses at critical target sites (Landínez-Macías et al., 2021). Therefore, the decrease in higher-order branches in axons expressing mutant FUS suggests that their connectivity is compromised. Combined with our *in vitro* data, this shows axonal defects may arise as early as the axon extension stage.

Our work builds on work showing nuclear FUS is essential for neuronal survival during development, by further supporting the hypothesis that cytoplasmic gain of function of mutant FUS compromises axonal development. As NLS mutation-induced motor neuron apoptosis can be rescued by FUS relocalisation to the nucleus in motor neurons only (Scekic-Zahirovic et al., 2016), it was suggested that cytoplasmic FUS localisation results in a toxic gain of function that autonomously affects motor neurons after differentiation, potentially via compromised formation of neuromuscular junctions (NMJs) and associated insufficient neurotrophin support (Scekic-Zahirovic et al., 2016). In this study, we observe morphological defects in neurons expressing the NLS mutant FUS(P525L) without loss of endogenous FUS, and using a FUS(16R) mutant that only partially mislocalises to the cytoplasm, which again points to a cytoplasmic gain of function mechanism. Our observation of axon looping further supports the hypothesis that defects in interactions with target cells are involved in FUS-induced developmental defects *in vivo*.

A potential mechanistic explanation for the phenotypes we observe may be altered axonal local protein synthesis (LPS). We have previously demonstrated that mutant FUS reduces growth cone protein synthesis in *Xenopus* RGCs (Murakami et al., 2015; Qamar et al., 2018). Given the increased levels of mutant FUS in *Xenopus* growth cones, this may arise due to reduced FUS granule dynamics affecting co-condensing RBPs and mRNAs (Qamar et al., 2018). These granule changes may affect direct FUS target mRNAs, but also target mRNAs of other axonal RBPs through sequestration or competition for binding, as has been proposed for FMRP (Birsa et al., 2021; Garone et al., 2021); however, it is challenging to study axonal translatome changes across multiple conditions using our model system. In addition, cytoplasmic FUS may induce non-local changes that affect LPS or axonal function generally, such as induction of stress responses (López-Erauskin et al., 2018).

Given the complexity of our model system, the phenotypes we observe may also be in part due to FUS-induced changes in non-RGC cell types, which may in turn affect LPS. In our *in vitro* cultures, as well as *in vivo*, it remains possible that RGC cell bodies experience changes in their local environment due to FUS expression in surrounding cells within the eye. In the *in vivo* experiments presented here, target tectal cells expressed mutant FUS, and so it remains possible that changes in tectal cell-derived cues contribute to the observed loss of axon complexity, potentially in addition to changes in RGC’s ability to respond to these cues. As target-derived cues act in part by modulating axonal protein synthesis (Lin et al., 2021), this could exacerbate axon-autonomous defects in LPS indued by mutant FUS. In addition, if the changes in growth cone stiffness that we observe are also present in actin-rich structures in other cells, resulting changes in the stiffness of nervous tissue overall would have non-cell-autonomous effects on axonal development *in vivo* (Gangatharan et al., 2018; Franze, 2020). In *Xenopus*, the brain area in which RGC axons branch (the optic tectum) is softer than the tissues through which the axons navigate, and this facilitates axon unbundling and therefore likely branching (Koser et al., 2016). Importantly, there is evidence that changes in nervous tissue stiffness occur in neurodegenerative disease and affect connectivity: brain stiffness correlates with default connectivity in AD patients (Murphy et al., 2015). The effects of such stiffness changes may selectively affect neurites, consistent with the axon-centric pathology of ALS: it has been reported that axons are considerably more mechanosensitive than cell bodies (Grevesse et al., 2015). Therefore, the role of cue-dependent branching and associated protein synthesis and the occurrence of (actin-associated) mechanical changes in ALS/FTD are important areas of future study.

Our findings regarding FUS-associated mechanical defects are relevant beyond the context of FUS-associated ALS, as changes in the cytoskeleton are a common feature of other variants of ALS/FTD. ALS and FTD have also been linked to mutations in cytoskeleton-associated genes, including in α-tubulin 4A (Smith et al., 2014; Mol et al., 2021), Spastin (Meyer et al., 2005; Münch et al., 2008), dynactin subunit-1 (Puls et al., 2003; Münch et al., 2004; Münch et al., 2005), kinesin family member 5A (KIF5A) (Nicolas et al., 2018; Saez-Atienzar et al., 2020), Profilin-1 (PFN1) (Wu et al., 2012) and Alsin (Hadano et al., 2001; Yang et al., 2001). While ALS has been repeatedly linked to changes in microtubules and associated axonal transport, less is known about changes in axonal actin, which are more challenging to study (Liu and Henty-Ridilla, 2022). Our work indicates dynamic growth cone actin is especially vulnerable to expression of mutant FUS, which is associated with mechanical defects. This hypothesis that mutant FUS affects actin is further supported by parallel work showing depolymerisation of both actin and tubulin networks for FUS(P525L)-expressing mammalian cells, which was also correlated with a reduction in apparent Young’s modulus (Chung et al., 2022). The generalisability of this finding to other forms of ALS/FTD is a subject for future research. Encouragingly in this regard, loss of the C9ORF72 protein results in reduced growth cone actin dynamics (Sivadasan et al., 2016).

The present study has limitations that mean these results should be validated in other model systems before they can be considered representative of changes to motor/cortical neurons that occur in ALS/FTD patients. Firstly, we use a human FUS overexpression system, which may introduce artefacts. These could occur due to functional differences between endogenous *Xenopus* FUS and human FUS, causing changes in RNA regulation. Furthermore, overexpression increases overall FUS levels, and it is known that aggressive ALS-like symptoms occur in transgenic mice overexpressing FUS(WT) (Mitchell et al., 2013). To control for this, we included a FUS(WT)-GFP condition in all our experiments, which we compare with a GFP only condition. We observed no toxicity of FUS(WT)-GFP expression. However, subtle changes in e.g. splicing profiles likely occur due to species-specific differences in FUS function or expression level changes, and so these results cannot be extrapolated to endogenous FUS in human neurons without further validation. Secondly, the present work has used RGCs as a model system of the axonal compartment, and future research is needed to determine the degree to which these findings are universal to different neuronal subtypes, particularly those most affected in ALS/FTD. RGCs are now known to be affected to an extent in ALS/FTD, albeit without onset of clear visual symptoms in patients (Rojas et al., 2020): loss of RGCs occurred in a model of advanced SOD1-assocciated ALS (Rojas et al., 2021), optic nerve thinning has been observed in FTD patients (Harrison et al., 2019), and neurofilament and TDP-43 inclusions occur in the retina of ALS patients (Fawzi et al., 2014; Sharma et al., 2020). Therefore, RGCs are not fully resistant in ALS/FTD, and are an informative model system to address fundamental questions regarding pathomechanisms. Some of these mechanisms likely apply to motor neurons, which may be more sensitive to their effects. For instance, as the NMJ may be especially vulnerable to changes in actin (Hensel and Claus, 2017), the changes in growth cone actin that we identify in RGCs likely affect NMJ assembly. Furthermore, motor neurons respond to similar sets of guidance cues as RGCs, including during the branching stage, and so RGC *in vivo* defects in cue-dependent transitioning to the branching stage of development may also be relevant for motor neurons. Future research in different model systems is needed to investigate the levels of these cues near the NMJ during development, as well as developing motor neurons’ ability to respond to them.

## Significance statement

This study demonstrates that mutation of the ALS/FTD (amyotrophic lateral sclerosis/frontotemporal dementia)-associated RNA-binding protein Fused in Sarcoma (FUS) can result in changes in axonal development. These changes occur both axon-autonomously in cytoskeletal organisation during axon extension and context-dependently during axonal branching. This indicates pre-symptomatic, developmental changes in axonal organisation may occur in familial disease variants.
